# Leaf Extracts of *Aerva lanata* Inhibit the Activities of Type 2 Diabetes-Related Enzymes and Possess Antioxidant Properties

**DOI:** 10.1155/2018/3439048

**Published:** 2018-09-26

**Authors:** Musbau Adewunmi Akanji, Samson Olasunkanmi Olukolu, Mutiu Idowu Kazeem

**Affiliations:** ^1^Department of Biochemistry, University of Ilorin, PMB 1515 Ilorin, Nigeria; ^2^Department of Biochemistry, Lagos State University, PMB 0001 Ojo, Lagos, Nigeria

## Abstract

The leaves of *Aerva lanata* are one of the indigenous medicinal plants used in the management of diabetes mellitus and its associated complications in Africa. However, its effect on the activities of diabetes-related enzymes has not been investigated. This study evaluated the *in vitro* inhibitory effects of different extracts of the *A. lanata* leaf on the activities of diabetes-related enzymes (*α*-amylase and *α*-glucosidase) and chemically induced free radicals. Aqueous, ethanol, and hydroethanol extracts of *A. lanata* leaves were subjected to a standard enzyme inhibition assay followed by determination of modes of inhibition of the enzymes. The antioxidant activities of the extracts were evaluated using 1,1-diphenyl-2-picrylhydrazyl (DPPH) and 2,2-azino-bis-(3-ethylbenzothiazoline-6-sulphonic acid) (ABTS). The results obtained showed that the hydroethanol extract of the *A. lanata* leaf optimally inhibited both *α*-amylase (IC_50_: 2.42 mg/mL) and *α*-glucosidase (IC_50_: 0.23 mg/mL). The Lineweaver-Burk plot revealed that the mode of inhibition of both enzymes by the hydroethanol extract was uncompetitive. However, the hydroethanol and aqueous extracts displayed the best DPPH and ABTS radical-scavenging ability, respectively. It can be concluded that the *A. lanata* extract inhibited the activities of both *α*-amylase and *α*-glucosidase uncompetitively, which may be attributed to its free radical-scavenging properties and rich phenolic composition.

## 1. Introduction

Diabetes is one of the global health emergencies of the 21^st^ century, which affects about 425 million people worldwide, and this may rise up to 629 million by the year 2045 [1]. Type 2 diabetes is the commonest form of this disease which is characterized by insulin resistance or reduced insulin sensitivity leading to hyperglycemia [2]. Management of type 2 diabetes normally entails lifestyle modification (diet and exercise) as well as treatment with oral hypoglycemic drugs, such as insulin secretagogues (e.g., glimepiride) to stimulate insulin secretion, biguanides (e.g., metformin) to decrease hepatic glucose output, thiazolidinediones (e.g., rosiglitazone) to improve insulin sensitivity, and alpha-glucosidase inhibitors (e.g., acarbose) to reduce starch and sucrose digestion [3].

Diabetes and some other diseases like cancer and stroke have been linked to oxidative stress which arises from the excessive production of free radicals in the mitochondrial electron transport chain [4]. Though the natural defence mechanism of animals detoxifies these free radicals with the aid of antioxidant molecules and enzymes, oxidative stress occurs when the effect of free radicals outweighs that of the cellular antioxidants [5]. Therefore, searching for antioxidant and antidiabetic agents from plants is an important strategy required to mitigate the widespread nature of diabetes. This is because present synthetic drugs have many drawbacks ranging from limited efficacy to several side effects such as hypoglycemia, weight gain, and chronic tissue damage.

*Aerva lanata* (Linn.) Juss. Ex Schult. (Amaranthaceae) is an erect or prostrate plant found in the tropical regions of Africa, India, Arabia, and the Philippines [6]. Commonly referred to as “ewe aje” in the western part of Nigeria and “polpala” in India, the plant enjoys extensive usage in traditional medicine. Different parts of the plant have been used in the treatment of several diseases including inflammation, malaria, kidney stone, rheumatism, bronchitis, haemorrhage, diuresis, jaundice, and diabetes [7]. The plant is very rich in phenolic compounds and alkaloids as well as steroids. Some of the isolated compounds include kaempferol, tiliroside, *β*-sitosterol, aervoside, syringic acid, and canthin-6-one [8]. A plethora of studies have reported the pharmacological potentials of *Aerva lanata* ranging from hepatoprotective, anti-inflammatory, antimicrobial, antihelminthic, and antitumour activities to antidiabetic activities [9].

Though there are reports on the hypoglycemic and antidiabetic potentials of *Aerva lanata*, there is no information on the inhibition of *α*-amylase and *α*-glucosidase by the plant. This study therefore presents inhibitory effects of the *Aerva lanata* extract on diabetes-related enzymes and free radical-scavenging properties of the plant.

## 2. Materials and Methods

### 2.1. Chemicals and Reagents

Porcine pancreatic *α*-amylase, rat intestinal *α*-glucosidase, 1,1-diphenyl-2-picrylhydrazyl (DPPH), quercetin, nitroblue tetrazolium (NBT), dinitrosalicylic acid (DNS), acarbose, and *para*-nitrophenyl-glucopyranoside (pNPG) were products of Sigma-Aldrich Co., St. Louis, USA, while starch soluble (extra pure) was obtained from J.T. Baker Inc., Phillipsburg, USA. Other chemicals and reagents were of analytical grade, and the water used was glass-distilled.

### 2.2. Plant Material

The leaf of *Aerva lanata* was obtained from the Igando area of Lagos in Nigeria in May 2013. It was identified and authenticated by Dr. A. B. Kadiri of the Department of Botany, University of Lagos, Nigeria, and a voucher specimen with reference number LUH 5600 was deposited in the university herbarium. The plant material was dried to constant weight in the laboratory at room temperature (22–25°C) and later grounded to powder using a laboratory blender.

### 2.3. Extract Preparation

Dried powdered material (30 g) was divided into three equal portions each weighing 10 g. Each portion was extracted in either 200 mL distilled water, ethanol, or hydroethanol (50 : 50) for 24 h. The extracts were centrifuged (Hermle Laboratory Centrifuge, Lasec, South Africa) and later filtered using Whatman No. 1 filter paper. The ethanol extract was concentrated to dryness using a rotary evaporator (Cole-Parmer, South Africa) under vacuum while the aqueous extract was freeze-dried in a lyophilizer (VirTis BenchTop, SP Scientific Series, USA). The hydroethanol extract was initially concentrated using a rotary evaporator and later freeze-dried in the lyophilizer. Extracts were dissolved in distilled water to prepare different concentrations (0.32, 0.63, 1.25, 2.5, and 5.0 mg/mL) of the extracts.

### 2.4. Antidiabetic Potentials

#### 2.4.1. *α*-Amylase Inhibitory Assay

A total of 250 *μ*L of each extract (0.32–5.0 mg/mL) was placed in a test tube, and 250 *μ*L of 0.02 M sodium phosphate buffer (pH 6.9) containing *α*-amylase solution was added. This solution was preincubated at 25°C for 10 min, after which 250 *μ*L of 1% starch solution in 0.02 M sodium phosphate buffer (pH 6.9) was added at timed intervals and then incubated at 25°C for 10 min. The reaction was terminated by adding 500 *μ*L of the dinitrosalicylic acid (DNS) reagent. The tubes were then incubated in boiling water for 5 min and cooled to room temperature. The reaction mixture was diluted with 5 mL distilled water, and the absorbance was measured at 540 nm using a spectrophotometer (Biowave II, Biochrom, UK) [10]. The control was prepared using the same procedure by replacing the extract with distilled water, while the activity of the standard was tested by replacing the extract with acarbose. The *α*-amylase inhibitory activity was calculated as percentage inhibition; thus,

(1) $$\begin{array}{c} \%inhibition=\left[\frac{{Abs}_{control}-{Abs}_{extract}}{{Abs}_{control}}\right]\times 100. \end{array}$$

#### 2.4.2. Mode of *α*-Amylase Inhibition

We followed the method of Kazeem and Ashafa [11] to determine the mode of inhibition of *α*-amylase by the plant extracts using the most potent extract. Briefly, 250 *μ*L of the (1.25 mg/mL) hydroethanol extract was preincubated with 250 *μ*L of *α*-amylase solution for 10 min at 25°C in one set of tubes. In another set of tubes, *α*-amylase was preincubated with 250 *μ*L of phosphate buffer (pH 6.9). 250 *μ*L of starch solution at increasing concentrations (25–400 *μ*g/mL) was added to both sets of reaction mixtures to start the reaction. The mixtures were then incubated for 10 min at 25°C and boiled for 5 min after the addition of 500 *μ*L of DNS to stop the reaction. The amount of reducing sugars released was determined spectrophotometrically using a maltose standard curve and converted to reaction velocities. A double reciprocal (Lineweaver-Burk) plot (1/*v* versus 1/[*S*]) where *v* is the reaction velocity and [*S*] is the substrate concentration was plotted to determine the mode of inhibition.

#### 2.4.3. *α*-Glucosidase Inhibitory Assay

Briefly, rat intestinal acetone powder (100 mg) was homogenized in 3 mL of 0.9% NaCl solution. After centrifugation (12000 ×g for 30 min), the crude enzyme (100 *μ*L) was incubated with 5 mM p-nitrophenyl glucopyranoside (pNPG) and 25 mM maltose or 50 mM sucrose in 0.1 M phosphate buffer (pH 6.9). This was followed by the addition of plant extracts (50 *μ*L) of different concentrations (0.32–5.0 mg/mL) to the mixture before incubation at 37°C for 30 min. The reaction was stopped by adding 50 *μ*L of 0.1 M Na_2_CO_3_. The enzyme activities were determined by measuring the absorbance at 405 nm (*α*-glucosidase) or 540 nm (maltase and sucrase) [12]. The control was prepared using the same procedure by replacing the extract with distilled water while the activity of the standard was tested by replacing the extract with acarbose. The percentage inhibition was calculated as follows:

(2) $$\begin{array}{c} \%inhibition=\left[\frac{{Abs}_{control}-{Abs}_{extract}}{{Abs}_{control}}\right]\times 100. \end{array}$$

#### 2.4.4. Mode of *α*-Glucosidase Inhibition

We followed the method of Kazeem and Ashafa [11] to determine the mode of inhibition of *α*-glucosidase by the extracts using the extract with the lowest IC_50_. Briefly, 50 *μ*L of the (1.25 mg/mL) aqueous extract was preincubated with 100 *μ*L of *α*-glucosidase solution for 10 min at 25°C in one set of tubes. In another set of tubes, *α*-glucosidase was preincubated with 50 *μ*L of phosphate buffer (pH 6.9). 50 *μ*L of PNPG, maltose, or sucrose at increasing concentrations (25–400 *μ*g/mL) was added to both sets of reaction mixtures to start the reaction. The mixtures were then incubated for 10 min at 25°C, and 500 *μ*L of Na_2_CO_3_ was added to stop the reaction. The amount of reducing sugars released was determined spectrophotometrically using a *para*-nitrophenol standard curve and converted to reaction velocities. A double reciprocal (Lineweaver-Burk) plot (1/*v* versus 1/[*S*]) where *v* is the reaction velocity and [*S*] is the substrate concentration was plotted to determine the mode of inhibition.

#### 2.4.5. Determination of IC_50_ Values of Antidiabetic Assays

The concentration of extracts or standard required to inhibit 50% of the enzyme concentration is known as IC_50_. IC_50_ values were determined from the percentage inhibitory capacities of the extracts using Microsoft Excel software.

### 2.5. Antioxidant Activities

#### 2.5.1. DPPH Free Radical-Scavenging Ability

Different concentrations (0.32–5.0 mg/mL) of the extracts (150 *μ*L) were mixed with 150 *μ*L of 0.4 mmol/L methanolic solution containing DPPH radicals. The mixture was left in the dark for 30 min, and the absorbance was measured at 516 nm using a microplate reader (Model 680, Bio-Rad). The DPPH free radical-scavenging ability of each extract was subsequently calculated with respect to the reference (which contains all the reagents without the test sample) [13]. The DPPH free radical-scavenging ability of the standard antioxidant was also tested by replacing the extract with quercetin.

#### 2.5.2. Superoxide Anion Radical-Scavenging Ability

Superoxide radicals were generated in 50 *μ*L of Tris-HCl buffer (16 mM, pH 8.0) containing 50 *μ*L of NBT (50 mM) solution, 50 *μ*L NADH (78 mM) solution, and different concentrations (0.32–5.0 mg/mL) of the extracts (100 *μ*L). The reaction started by adding 1 mL of phenazine methosulphate (PMS) solution (10 mM) to the mixture. The reaction mixture was incubated at 25°C for 5 min, and the absorbance was measured at 560 nm in a microplate reader (Model 680, Bio-Rad, USA) [14]. The superoxide anion radical-scavenging ability of the standard antioxidant was also tested by replacing the extract with quercetin.

#### 2.5.3. Determination of EC_50_ Values of Antioxidant Assays

The concentration of extracts or standard required to scavenge 50% of free radicals or chelate metal ions is known as EC_50_. EC_50_ values were determined from the free radical-scavenging or iron-chelating abilities of the extracts using Microsoft Excel software.

### 2.6. Statistical Analysis

Statistical analysis was performed using the GraphPad Prism 5 statistical package (GraphPad Software Inc., La Jolla, CA, USA). The data were analyzed by one-way analysis of variance (ANOVA) followed by the Bonferroni test. All the results were expressed as mean ± SEM for triplicate determinations.

## 3. Results

**Figure 1:**
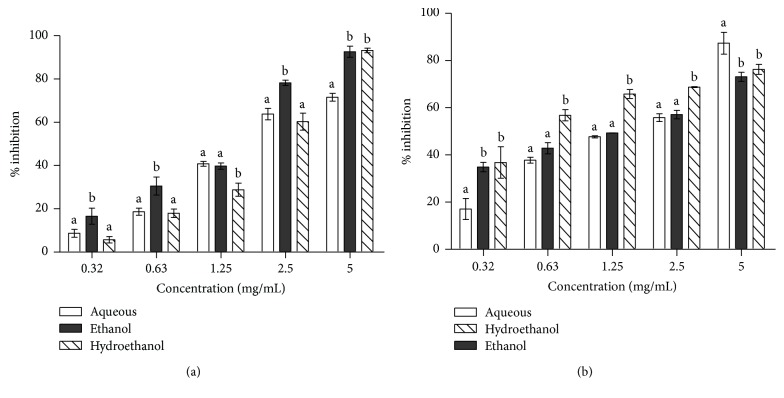
Inhibitory effects of leaf extracts of *Aerva lanata* on the activities of (a) *α*-amylase and (b) *α*-glucosidase. Bars carrying different letters at the same concentration are significantly different (*p* < 0.05).

Figure 1 shows the inhibitory effects of different extracts of *Aerva lanata* on the activities of *α*-amylase and *α*-glucosidase. At lower concentrations (0.32–0.63 mg/mL) and 2.5 mg/mL, the ethanol extract displayed significantly higher inhibition of *α*-amylase compared to the other extracts (Figure 1(A)). However, at concentrations 1.25 and 5.0 mg/mL, the hydroethanol and aqueous extracts, respectively, displayed significantly lower inhibition of the enzyme. At the lowest (0.32 mg/mL) and highest concentrations (5.0 mg/mL), the percentage inhibition of *α*-glucosidase by the aqueous extract is significantly lower and higher, respectively, compared to the other extracts (Figure 1(B)). In between these concentrations, the hydroethanol extract displayed significantly higher inhibition of the enzyme compared to other extracts.

Table 1 shows the IC_50_ for the inhibition of the activities of *α*-amylase and *α*-glucosidase by the different extracts of *Aerva lanata*. As for *α*-amylase, the ethanol extract displayed the lowest IC_50_ compared to the other extracts but was similar to the standard, acarbose. On the other hand, the hydroethanol extract displayed the lowest IC_50_ for the inhibition of *α*-glucosidase compared to all the extracts and the standard.

**Figure 2:**
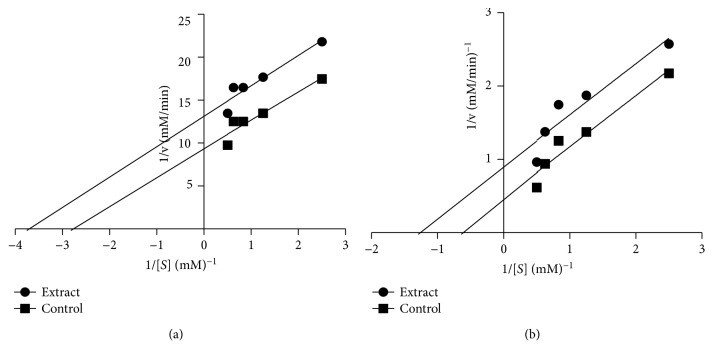
Mode of inhibition of (a) *α*-amylase and (b) *α*-glucosidase by the hydroethanol extract of the *Aerva lanata* leaf.

The mode of inhibition of the enzymes by the hydroethanol extract of *Aerva lanata* is shown in Figure 2. Figures 2(a) and 2(b) depict that the hydroethanol extract of *Aerva lanata* inhibited both *α*-amylase and *α*-glucosidase in an uncompetitive manner.

**Figure 3:**
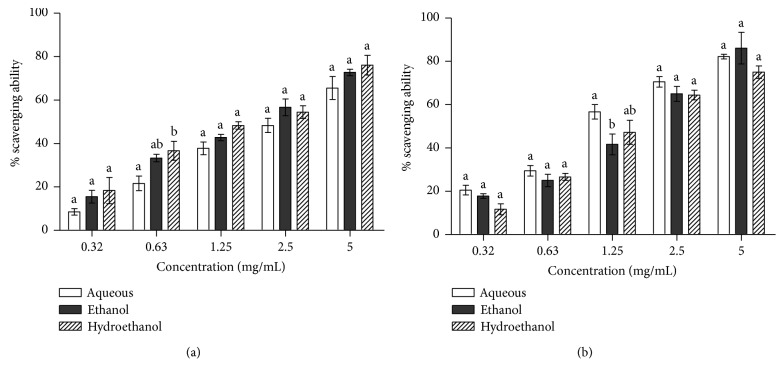
(a) DPPH and (b) ABTS radical-scavenging abilities of leaf extracts of *Aerva lanata*. Bars carrying different letters at the same concentration are significantly different (*p* < 0.05).

Figure 3 shows the result of the DPPH and ABTS radical-scavenging abilities of different extracts of the *Aerva lanata* leaf. Though there are variations, there is no significant difference in the DPPH radical-scavenging abilities among all the extracts tested except for the concentration of 0.63 mg/mL where the aqueous extract is significantly different from the hydroethanol extract (Figure 3(A)). A similar trend is also reflected in the ABTS radical-scavenging abilities where the scavenging abilities of the extracts are similar except at the concentration of 1.25 mg/mL in which the activities of the aqueous extract of the plant are significantly higher than those of the ethanol extract.

Table 2 presents the EC_50_ for the free radical-scavenging abilities of the various extracts of *Aerva lanata*. Among all the extracts, the hydroethanol extract exhibited the lowest EC_50_ for the DPPH radical-scavenging ability but was similar to the ethanol extract and significantly higher than the standard, gallic acid. Conversely, the aqueous extract of *Aerva lanata* displayed the lowest EC_50_ for the ABTS radical-scavenging abilities compared to the extracts and the standard. However, the EC_50_ for free radical-scavenging abilities exhibited by both ethanol and hydroethanol extracts are similar.

The phenolic and flavonoid composition of various extracts of *Aerva lanata* is shown in Table 3. The hydroethanol extract possessed the highest quantity of phenolics followed by the aqueous extract. However, the ethanol extract is the richest in flavonoids compared to the other extracts.

## 4. Discussion

We investigated the antidiabetic and free radical-scavenging properties of different extracts of *Aerva lanata* using *in vitro* models. Its antidiabetic properties was assessed by testing for its inhibitory effect on *α*-amylase and *α*-glucosidase, while the antioxidant activities were investigated by evaluating its ability to scavenge DPPH and superoxide radicals.

Inhibition of enzymes involved in the hydrolysis of carbohydrates such as *α*-amylase and *α*-glucosidase is one of the therapeutic approaches for diabetes-related hyperglycemia [3]. Pancreatic *α*-amylase is involved in the conversion of starch into disaccharides and oligosaccharides while intestinal *α*-glucosidase catalyzes the breakdown of disaccharides into glucose [15]. Inhibition of these enzymes would slow down the degradation of starch in the gastrointestinal tract, thereby ameliorating hyperglycemia.

The ethanol extract of *Aerva lanata* displayed the strongest inhibition of *α*-amylase which culminated in the lowest IC_50_ when compared to other extracts and acarbose. This is undesirable of a good hypoglycemic agent as excessive inhibition of the enzyme is responsible for drawbacks associated with acarbose such as abdominal distention and hypoglycemia [16]. The hydroethanol extract on the other hand exhibited the strongest inhibition of *α*-glucosidase and possessed the lowest IC_50_ for the inhibition of the enzyme. Taken together, the hydroethanol extract of *Aerva lanata* seems to be the most potent inhibitor of both enzymes due to its mild inhibition of *α*-amylase and strong inhibition of *α*-glucosidase. This is in conformity with previous reports that a desirable antidiabetic agent from a plant should be a mild inhibitor of *α*-amylase and strong inhibitor of *α*-glucosidase [10, 14].

In order to determine the modes of inhibition of the enzymes by *Aerva lanata*, the most potent extract (hydroethanol) of the plant was chosen for the study. The Lineweaver-Burk plot revealed that the hydroethanol extract of *Aerva lanata* inhibited both *α*-amylase and *α*-glucosidase in an uncompetitive manner. This implies that the active component in the extract only recognizes and binds to the enzyme-substrate (ES) complex, without binding to the free enzyme [17]. This type of inhibition is characterized by a reduction in both substrates K_m_ and V_max_, and the inhibitor binds to the enzyme target only when the target is active and the substrate is present [18]. This may be useful in the design of *α*-amylase and *α*-glucosidase inhibitors for the treatment of diabetes mellitus.

Oxidative stress has been implicated in the pathogenesis of several diseases including diabetes mellitus. Previous studies also showed that hyperglycemia induces the generation of free radicals, which aggravates the development of diabetes and its associated complications [19, 20]. This is why the antioxidant potential of the *Aerva lanata* extracts was assessed to ascertain the relationship between antidiabetic and antioxidant potentials of the plant. The hydroethanol extract of the plant displayed the lowest EC_50_ for the scavenging of DPPH radicals, which implies that it possessed the best DPPH radical-scavenging abilities. On the contrary, the aqueous extract of the plant exhibited the lowest IC_50_ for scavenging of superoxide radical (ABTS). The superoxide radical is implicated in several diseases because it plays an important role in the formation of other reactive oxygen species such as hydroxyl radical and singlet oxygen, which causes oxidative damage to biomolecules [21, 22].

In consonance with the outcome of the *in vitro* hypoglycemic and antioxidant abilities of *Aerva lanata* extracts, compositional analysis revealed that the hydroethanol extract is the richest in terms of phenolics. Phenolic compounds are one of the most numerous and widely distributed secondary metabolites in plants [23]. They possess a wide range of pharmacological activities including antimicrobial, antidiabetic, anticancer, anti-inflammatory, and antithrombotic activities [24]. All these functions have been attributed to their chemical composition and antioxidant properties. It can therefore be suggested that the antidiabetic activities of the *Aerva lanata* extracts may be due to the phenolic composition of the plant and its antioxidant activities.

In conclusion, the aqueous, ethanol, and hydroethanol extracts of the *Aerva lanata* leaf inhibited the activities of diabetes-related enzymes and possessed free radical-scavenging abilities. Out of the three extracts, the hydroethanol extract displayed the most effective antidiabetic as well as antioxidant potential. This may be due to its high phenolic content.

## Figures and Tables

**Table 1:** IC_50_ values for in vitro *α*-amylase and *α*-glucosidase inhibition by various extracts of *Aerva lanata* and acarbose.

Extracts	IC_50_ (mg/mL)	
	*α*-Amylase	*α*-Glucosidase
Aqueous	2.66 ± 0.15^a^	2.01 ± 0.12^a^
Ethanol	1.85 ± 0.03^b^	1.75 ± 0.05^b^
Hydroethanol	2.42 ± 0.05^a^	0.23 ± 0.02^c^
Acarbose	2.10 ± 0.07^b^	1.63 ± 0.10^b^

**Table 2:** EC_50_ values for the free radical-scavenging activities of different extracts of *Aerva lanata* leaves.

Extract	EC_50_ (mg/mL)	
	DPPH	ABTS
Aqueous	3.18 ± 0.04^a^	1.79 ± 0.06^a^
Ethanol	2.48 ± 0.01^b^	2.14 ± 0.10^b^
Hydroethanol	2.25 ± 0.04^b^	2.35 ± 0.02^b^
Gallic acid	1.25 ± 0.02^c^	2.03 ± 0.02^c^

**Table 3:** Total phenolic and flavonoid composition of *Aerva lanata* leaves.

Extract	Phenolics (mg/g)	Flavonoids (mg/g)
Aqueous	55.91 ± 2.75^a^	7.89 ± 0.11^a^
Ethanol	30.72 ± 3.01^b^	17.67 ± 0.25^b^
Hydroethanol	78.15 ± 2.50^c^	14.30 ± 0.40^b^

## Data Availability

The data used to support the findings of this study are included within the article.
